# Administration Route Differentiation of Altrenogest via the Metabolomic LC-HRMS Analysis of Equine Urine

**DOI:** 10.3390/molecules29214988

**Published:** 2024-10-22

**Authors:** Madysen Elbourne, John Keledjian, Adam Cawley, Shanlin Fu

**Affiliations:** 1 Centre for Forensic Science, University of Technology Sydney, Sydney, NSW 2007, Australia; madysen.a.elbourne@student.uts.edu.au; 2 Australian Racing Forensic Laboratory, Racing NSW, Sydney, NSW 2000, Australia; johnk@racingnsw.com.au; 3 Racing Analytical Services Limited, Flemington, VIC 3031, Australia

**Keywords:** altrenogest, metabolomic analysis, LC-HRMS, equine urine, androgenic anabolic steroids

## Abstract

Altrenogest, also known as allyltrenbolone, is a synthetic form of progesterone used therapeutically to suppress unwanted symptoms of estrus in female horses. Altrenogest affects the system by decreasing levels of endogenous gonadotrophin and luteinizing and follicle-stimulating hormones, which in turn decreases estrogen and mimics the increase of progesterone production. This results in more manageable mares for training and competition alongside male horses while improving the workplace safety of riders and handlers. However, when altrenogest is administered, prohibited steroid impurities such as trendione, trenbolone, and epitrenbolone can be detected. It has been assumed that greater concentrations of these steroid impurities are present in injectable preparations and, therefore, pose a greater risk of causing anabolic effects when administered. For this reason, and due to the necessity of this therapeutic substance for the safety of thoroughbred racing participants, a metabolomic approach investigating the differentiation of two main administration routes was conducted. Liquid chromatography high-resolution mass spectrometry analysis of equine urine samples found five sulfated compounds, estrone sulfate, testosterone sulfate, 2-methoxyestradiol sulfate, pregnenolone sulfate, and cortisol sulfate, with the potential to differentiate between oral and intramuscularly administered altrenogest using a random forest classification model. The best model results, comparing two horses’ administration normalized peak area datasets, gave an AUC score of 0.965 with a confidence level of 95% (between 0.931 and 0.995). Identifications of these compounds were confirmed with assistance from the Shimadzu Insight Explore Assign feature, together with MS/MS spectrum and retention time matching of purchased and synthesized reference standards. This study proposes a new potential application for metabolomic multi-tool workflows and machine learning models in a forensic toxicological context.

## 1. Introduction

Anabolic androgenic steroids (AAS), categorized as synthetic derivatives of the endogenous steroid hormone testosterone, are some of the most common illegal compounds used in the sporting industry [1,2,3,4]. AAS have substantial effects on the body, including increased stamina and muscle tone, which may benefit athletic performance [5]. The use of these compounds is prohibited in equine sports due to their performance-enhancing benefits [6]. 

Altrenogest (ALT), also known as allyltrenbolone, is a synthetic form of progesterone used therapeutically to suppress unwanted symptoms of estrus in female horses [7,8]. ALT affects the system by decreasing levels of endogenous gonadotrophin hormones and luteinizing and follicle-stimulating hormones, which in turn decreases estrogen and mimics the increase of progesterone production [9]. This results in more manageable mares for training and competition alongside male horses while improving the workplace safety of riders and handlers [10]. However, when ALT is administered, prohibited AAS impurities, including trendione (as a major impurity), trenbolone (minor), and epitrenbolone (from metabolism), can be detected [7,11,12]. Additionally, it has been anticipated that higher concentrations of these AAS impurities are present in intramuscularly (IM) injected forms of the compound in comparison to the oral forms available to thoroughbred veterinarians and trainers [13]. For this reason, some racing bodies have discouraged the use of injectable ALT and allow orally administered ALT only outside one clear day of racing [13,14].

Detection of steroids of either endogenous or exogenous origin has been frequently performed by measuring free steroids following enzymatic hydrolysis of equine urine [15]. It is known that enzyme-catalyzed hydrolysis is less effective in hydrolyzing the phase II steroid sulfate conjugates [15,16], leading to possible inaccurate detection of some steroid metabolites. To overcome the complexity associated with hydrolysis techniques for urinary AAS [17], advances in liquid chromatography-mass spectrometry (LC-MS) have enabled direct analysis of intact phase II conjugated metabolites [18].

The development of a complementary approach has the potential to improve injected ALT detection, using features differing from oral ALT. Abnormal levels of endogenous substances can provide an extended window of detection and give rise to the possibility of a biomarker or biomarker ratio to distinguish between oral and IM administration [19]. LC-MS was utilized to analyze intact phase II conjugates without derivatization. Sulfate conjugate analysis was the focus of this research, due to the nature of excretion of these steroids of interest, particularly relevant to equine metabolism [15,16,20].

Additionally, recent advancements in machine learning tools and multi-tool workflows for the processing and analysis of high throughput mass spectrometry data have enabled a more advanced investigation of metabolomics studies. This has led to improved/streamlined outcomes for biomarker discovery and anti-doping detection in the clinical and toxicological fields [21,22]. This study adopted some of these novel tools to assess their benefit discerning between two administration scenarios (oral and IM). Whilst there has been substantial research using these methods to discern the presence or absence of drug administration [23], their potential use to differentiate between two administration routes of the same substance, at similar concentrations, does not appear to be as well explored. 

This research focused on an untargeted screening of the metabolomic profile in mares administered with ALT. The primary aim was to identify potential biomarkers that could assist in distinguishing oral administration from the IM route. An untargeted metabolomic approach was utilized to monitor urinary levels of AAS and other metabolically related endogenous and exogenous steroids as both phase I (unconjugated) and phase II (sulfate conjugates), where possible.

## 2. Results

### 2.1. MS Annotation and Statistical Analysis

**Figure 1:**
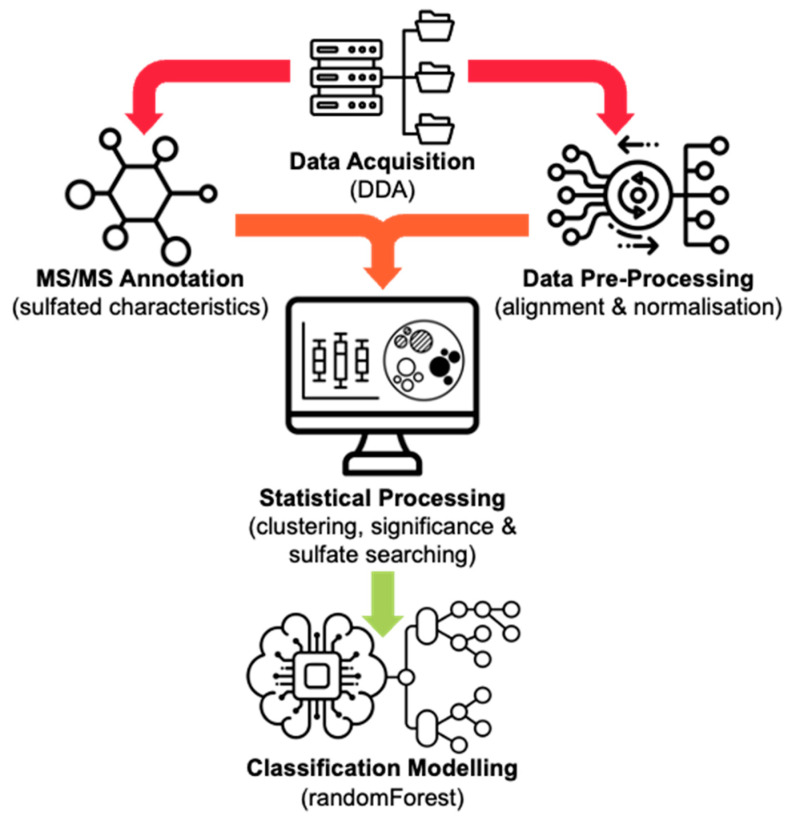
An overview of the presented metabolomic data processing workflow. Icons sourced from Noun Project©.

Metabolomic data was acquired using ultra-high-performance liquid chromatography high-resolution mass spectrometry (UHPLC-HRMS/MS, Shimadzu, Kyoto, Japan) with data-dependent acquisition (DDA) following solid phase extraction (SPE) sample clean-up of equine urine samples collected from mare horses administered with ALT, either orally (*n* = 3) or intramuscularly (*n* = 3). Succeeding data acquisition, raw Shimadzu LC files were exported and input into MS-DIAL, where the MS data underwent data alignment and locally weighted scatterplot smoothing (LOWESS) normalization of total ion chromatograms (TIC) by applying tolerances to both retention time (RT) (±3 s) and mass accuracy (Δ*m*/*z* ± 5 ppm), alongside the recommended settings for DDA-HRMS systems [24]. Jupyter Notebook was used to read two custom Python scripts that extracted and appended MS/MS data for each aligned feature containing a sulfate-derived fragment (negative ions *m*/*z* 79.9573 (SO_3_^−^), 80.9652 (HSO_3_^−^), 95.9523 (SO_4_^−^), 96.9601 (HSO_4_^−^), and neutral losses 79.9568 Da (SO_3_) and 97.9674 Da (H_2_SO_4_)), before realigning it to the MS-DIAL normalized peak area output. A custom R script, run through RStudio, was then used to conduct statistical analysis. This started with a *k*-means clustering function to sort metabolic features [25]. Clustering was performed based on the normalized data of eight unique parameters, all derived from the MS/MS spectral information. These included the six characteristic sulfate-derived fragments, as mentioned above, as well as two calculated parameters, i.e., intensity ratio (IR), the total sum of sulfate-derived fragments divided by the sum of all fragments, and maximum abundance (MA), the normalized relative abundance (%) of the largest sulfate-derived fragment. This allowed clustering to occur into non-sulfate and sulfate groupings due to the abundant nature of steroid sulfate fragmentation peaks (Figure 1). 

High throughput differential metabolite level analysis and data visualization was then performed in R using the *limma* and *ggplot2* packages, respectively. The output gives a final curated list of metabolic features with associated statistical results, UHPLC-HRMS/MS data (RT and *m*/*z*), and product ion data for any sulfate-derived fragments detected by MS/MS. A novel steroid sulfate searcher function, coded into the R script, that extrapolated potential steroid sulfate features from the dataset was also used. 

**Figure 2:**
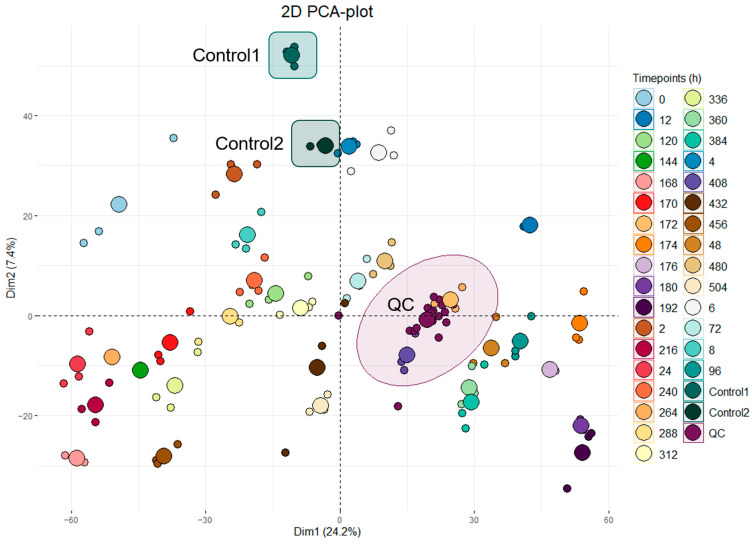
A 2-dimensional ellipses PCA plot containing a representative dataset from horse 6 with 32 time points (h) of urine samples analyzed in technical triplicates. Pooled QC samples are noted in burgundy/purple, and their clustering is indicated with a circle. Control 1 and Control 2 samples are colored and circled in turquoise green and deep green, respectively.

Two-dimensional ellipses PCA plotting was conducted on all analyzed batches. Figure 2 shows a representative dataset from one of the IM-administered horses with 32 sequential time points (0–504 h) of urine samples analyzed in technical triplicates. Pooled QC samples, prepared by combining a small amount of each sample from a single administered horse, can be seen in burgundy/purple, and their close clustering is indicated with an ellipse (labeled ‘QC’). Two other sample groups are coincidentally also included in the cluster but considered unrelated. Two external control samples (Control 1 and Control 2, colored and circled in turquoise green and deep green, respectively) were also analyzed alongside each batch using pre-administration (T0) samples from other horses in the study.

**Figure 3:**
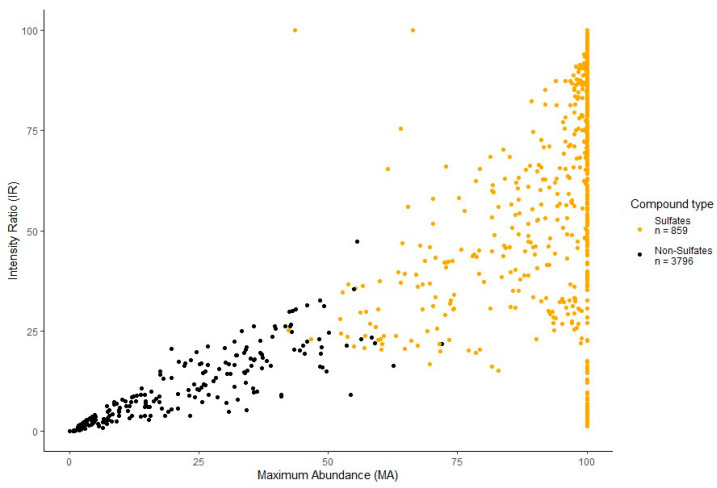
A *k*-means clustering scatterplot separating sulfated (orange dots, *n* = 859) and non-sulfated (black dots, *n* = 3796) features labeled during data processing. Separation of features is defined by the maximum abundance (MA) and intensity ratio (IR) calculated during the pre-processing of the dataset.

The *k*-means clustering of the data is represented by the scatterplot (Figure 3) separating sulfated (orange dots, *n* = 859) and non-sulfated (black dots, *n* = 3796) features labeled during data pre-processing. Separation of features occurs due to the predictability of sulfated fragmentation and is well represented by the calculated characteristics MA and IR. As mentioned above, high values of MA and IR for a feature imply a higher likelihood of a sulfated feature, whereas lower values imply the opposite. From here, each feature is assigned a cluster label 1 or 2, indicating sulfated or non-sulfated, respectively, to assist in the separate analysis of sulfated features in the proceeding steps.

**Figure 4:**
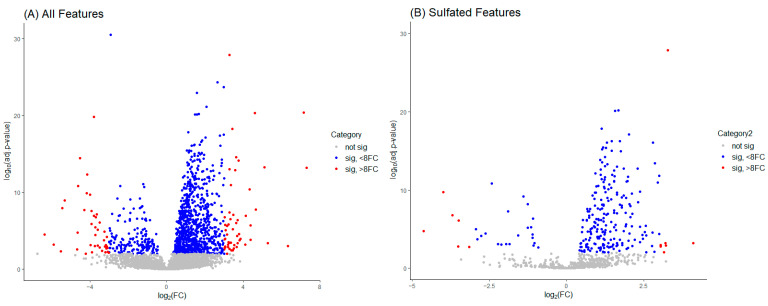
Volcano plots for visual identification of significantly changed features in the metabolic dataset. Plot (**A**) is inclusive of all features identified in the pre-processing of the data, whilst plot (**B**) only concerns sulfate-labeled data points.

Volcano plots aid the visual identification of significantly changed features (from pre-administration (0 h) samples) in the metabolic dataset. In Figure 4, plot A includes all features identified by MS-DIAL in the pre-processing of the data, whilst plot B only displays sulfate-labeled data points. Non-significant values denoted in grey have a log-transformed adjusted *p*-value of less than 1. Blue-colored features have an adjusted *p*-value greater than 1 and a fold change (FC) value less than ±8 (adjusted log FC less than ±3) and are classified as mid-significant. Red-colored features are considered significant with an FC value greater than ±8 (adjusted log FC greater than ±3). 

In this example (Figure 4), plot A identifies 3567 non-significant, 993 mid-significant, and 95 significant features. The significant features are inclusive of 41 down-regulated (FC > −8) and 54 up-regulated (FC > +8) features of interest. Plot B shows 615 non-significant, 232 mid-significant, and 13 significant features, with the significant grouping containing 6 down-regulated and 7 up-regulated features for further investigation.

The *m*/*z* of all sulfate-labeled features were then compared to a predetermined list of theoretical steroid sulfate compounds (26 in total), mostly endogenous and present in the steroid hormone metabolism pathway. From the 859 sulfated features extracted from the *k*-means scatterplot, the ‘if else’ search function identified 31 potential features that had a Δ*m*/*z* within ±5 ppm to 14 of the listed metabolites. It is worth noting that while multiple features can be preliminarily allocated the same theoretical metabolite, manual screening of the output was then conducted to investigate collected MS/MS and retention time for isolation of most-likely true identification. This manually filtered list of theoretically identified features was then compared across all administration horses to account for consistent metabolite presence. This gave confidence that the selected compounds for classification modeling would be reliably identifiable in any unrelated urine sample. 

Table 1 presents a combined list of selected potential sulfate-conjugated steroid metabolites extracted from the steroid sulfate searcher function, as explained above. The volcano plot results of these features were considered during the selection process; however, some more up-regulated features from the plots were omitted due to the lack of consistent appearance in all six horse administrations or their non-steroid-like structure, as indicated by MS data, including MS/MS fragmentation. 

### 2.2. Compound Identification

Verification of feature metabolite identification is necessary to proceed with the biomarker proposal/investigation. Shimadzu’s Insight Explore Assign feature was utilized to gain confidence in preliminary identification assumptions before reference standards were purchased and/or synthesized (provided by C.C.J. Fitzgerald [26,27]) for MS/MS fragmentation and retention time matching. A captured spectrum from an administration sample was compared to the theoretical fragmentation of 1000 potential compounds with matching *m*/*z*, extracted from ChemSpider, and returned a similarity score report for these potential identifiers based on the proposed MS/MS fragmentation. Mass accuracy tolerance could be adjusted and was set at Δ*m*/*z* ± 5 ppm for this investigation. From Table 1, features (1), (2), (3), (4) and (5) were putatively identified as estrone sulfate (E1S), testosterone sulfate (TS), 2-methoxyestradiol sulfate (2-ME2S), pregnenolone sulfate (PregS), and cortisol sulfate (CS), respectively (Table 2), using the steroid sulfate searcher function, and in Assign, returned convincing similarity reports to corresponding proposed MS/MS fragmentation. 

Whilst Assign is beneficial in providing an indication of potential compound similarity from unknowns, these results are hypothesis-based and require further investigation with reference standards to confirm results. Synthesized reference standards of some previously mentioned features, E1S (1), TS (2), and PregS (4), were obtained and analyzed in matching conditions on the same instrument to determine true *m*/*z*, RT, and likely fragmentation patterns (Section S1). Compounds 2-ME2S (3) and CS (5) still require synthesis to complete the verification of compound identification. 

### 2.3. Classification Modeling

The use of a random forest (RF) classification model was tested with the five mentioned features, comparing one oral (*n* = 90) horse dataset and one IM (*n* = 93) horse dataset, and resulted in strong differentiation between the oral and IM administration routes. This combined dataset totaled 183 normalized peak area data points incorporating 61 post-administration urine samples (i.e., time zero/pre-administration samples removed), analyzed as technical triplicates. The non-uniform sample size per horse is due to the orally administered horses’ missing time point of 480 h. All model types were trialed in MetaboAnalyst’s Biomarker Analysis tool, including linear support vector machines (SVM) and partial least squares–discriminant analysis (PLS-DA) in addition to RF. However, RF was found to be the most successful, with higher prediction accuracy results compared to SVM and PLS-DA.

These five potential markers, E1S, TS, 2-ME2S, PregS, and CS, were selected based on their consistent presence in the equine steroid hormone metabolic pathway and the direction of log_2_-fold change values calculated in the previous statistical analysis. Within MetaboAnalyst, the dataset was filtered for any missing values (which, by default, were replaced with a value of 20% of the minimum peak intensity recorded for that feature). No data scaling was applied. Data transformation was also not selected due to the model type (RF) used in this study. Further details of the MetaboAnalyst workflow can be found in the Supplementary Materials (Section S2). 

**Figure 5:**
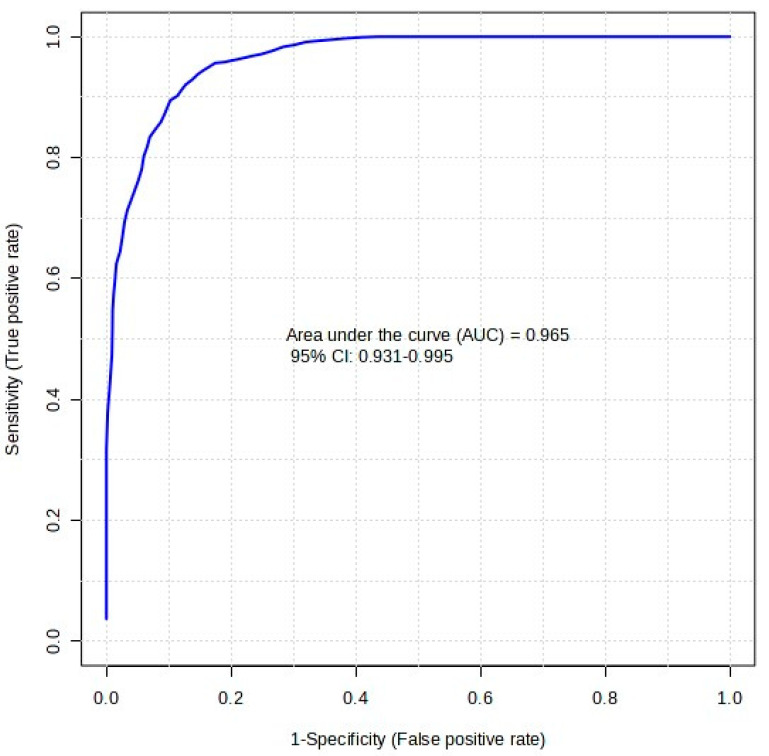
ROC curve plot for a random forest analysis of five biomarkers of interest. The dark blue line is inclusive of all five features, giving an AUC score of 0.965 with a confidence level of 95% (between 0.931 and 0.995).

Table 3 presents the classical univariate analysis of each compound and its individual biomarker potential. Of the five features, 2-ME2S produced the highest area under the curve (AUC) result of 0.840. This AUC score implies that, on its own, 2-ME2S would provide reasonable classification between oral and IM samples. The inclusion of all five features in a multivariate RF model, however, allows for greater robustness in the model. This is shown by the receiver operating characteristics (ROC) curve in Figure 5. The dark blue line in the plot is inclusive of all five features and gave an AUC score of 0.965 with a confidence level of 95% (between 0.931 and 0.995). This translates to a predictive accuracy of 86.7%. This ROC curve is generated through Monte-Carlo cross validation (MCCV), where 66.6% (two-thirds, 2/3) of the dataset are used to evaluate feature importance (i.e., train) in the model, and the remaining 33.3% (one third, 1/3) of the data points are used to validate the model [28,29]. The ranking method used by MetaboAnalyst in this model was RF, ‘mean decrease in accuracy’, with a subsampling (nRuns) value of 30. The number of trees created in this RF model was set at a default of 300. Reference [30] provides more information on the RF model implemented in MetaboAnalyst and links to the open-access GitHub© page with MetaboAnalystR 3.0 script.

From 93 IM samples and 90 oral samples (183 total samples), the model was able to correctly identify IM administration in 83 out of 93 samples (Table 4). This correlates to a true positive result of 89%. Likewise, oral administration was accurately identified 75 times out of 90 occurrences, which corresponds to a true negative score of 83%.

## 3. Discussion

The RF model presented above, involving five biomarkers, E1S, TS, 2-ME2S, PregS, and CS, showed promising classification of IM ALT administration distinct from oral administration of ALT. The selected group of biomarkers may be considered advantageous in targeting various areas of endogenous steroid metabolism and exogenous steroid excretion and influence. This work should be considered a preliminary study with further validation required from additional administration studies and/or equine athlete (race day) sampling scenarios. Additionally, further verification of 2-ME2S and CS compound identity is required. 

RF modeling, developed by Leo Breiman in 2001, is a group of regression trees made from the random selection of samples of the training data [31,32]. According to previous literature, RF has the advantages of high prediction classification accuracy, strong generalization, and fast training speed, and can be used for classification, regression, and feature importance analysis. RF is often considered a more robust tool when compared to other similar tree-based classifier models (decision trees (DT), also known as classification and regression trees (CART)). Recently, RF has become popular as a biomarker detection tool in various metabolomics studies as it boasts the strength to deal with missing data and overfitting issues [33,34]. Additionally, it can also tackle high-dimensional data sets without requiring feature elimination [34]. 

There are many previously discussed benefits of implementing a multi-tool workflow into metabolomic study for biomarker discovery [35]. The primary goal in this scenario is to provide a preliminary screening tool of the collected metabolomic data for markers of interest. Additionally, these tools automate tedious and time-consuming tasks when processing and statistically analyzing an extensive dataset [35]. Incorporating a workflow also allows the ability to target a specific area of the metabolomic pathway, if this is desired, with relative ease. With the increased popularity of this processing and analysis technique over the last decade, additional tools have become available to a wider research demographic with open-source accessibility, as well as increased customization of already published script-based tools for optimization to a specific research question [26]. In relation to the dataset presented above, MS-DIAL, custom Python and R scripts, and MetaboAnalyst formed the multiplatform workflow utilized to acquire the presented results.

After the initial screening of metabolomic data, as demonstrated in this paper, it is necessary to verify the identity of the potential biomarker, or group of markers, extracted. There is a range of tools available to assist at this stage of putative identification before final confirmation with purchased reference standards. MS spectrum databases such as Human Metabolome Database (HMDB-https://hmdb.ca, accessed on 13 August 2024), MassBank of North America (MoNA-https://massbank.us, accessed on 13 August 2024), ChemSpider (https://chemspider.com, accessed on 13 August 2024), and NIST Chemistry WebBook (https://webbook.nist.gov/chemistry, accessed on 13 August 2024) databases are all easily accessible and relatively user-friendly resources. MS annotation tools are also an alternative that investigates the untargeted dataset directly, typically to extract fragmentation patterns relating to a subset of compounds predetermined by the user [36]. This study utilized the Assign feature offered by Shimadzu to add weight to the suspected identification of features before purchasing and synthesizing the appropriate reference standards for confirmation.

Identified compounds should then be subjected to robustness testing to assess their suitability as a biomarker in the desired context. Consistency and reproducibility in their presence or absence in appropriate samples (influenced by oral or IM administrations) and detectable peak intensity are necessary for a successful and reliable biomarker [36,37]. While this group of steroids has been proposed as having the potential to discriminate between an oral and IM administration of altrenogest in mares, this trial was conducted on a limited dataset of administration horses. Ultimately, additional testing is required before implementation would be possible. Such testing includes application to race day samples collected from active racehorses, as it has been previously established that it is possible for athletic horses to have differing steroid excretion profiles to stationary/grazing horses [38].

The use of a group of biomarkers is largely considered more favorable over a single marker detection or a biomarker ratio of two features. The adoption of a multivariate analysis over a uni- or bivariate presents some advantages to solving complex issues, due to its own more complex nature [35]. This does, however, also act as a limitation to analysts with beginner or intermediate computational and coding skills, which, in developing, can cost the user large amounts of time, especially when grasping more “complex” statistical/machine learning and modeling tools. Additionally, the choice of biomarkers identified in this study provides a broad overview of the excreted urinary steroid hormone metabolism pathway previously acknowledged in the equine species [26].

Previously, research has focused on the anabolic and androgenic nature and pharmacodynamic effects of ALT on male, female, and gelded horses [11,39]. However, that has not addressed the detection of ALT based on the type of administration route, which has recently become a focal point in the equine industry. The differentiation between ALT administration types from an excreted urine sample will greatly benefit the thoroughbred racing industry, due to the nature of race day sampling, ALT as an allowed therapeutic substance, and the complicated situation regarding the steroid impurities present in commercial products.

### Study Limitations and Future Work

A fluctuation in the number of steroid sulfates captured with the coded steroid sulfate search function and in prior *k*-means clustering could indicate variability in the number of features captured between administration horses and should be considered a limitation of this study. This is most likely due to any small sample preparation variability or a change in wait time between extraction and instrument availability (due to instrument failures), for which extracted samples were stored at 4 °C until they were able to be analyzed. 

This work should be considered a preliminary study with further validation required from external administration studies and/or equine athlete (race day) sampling scenarios. Ideally, this model needs to be verified with samples unrelated to the batch used to identify the five chosen features. Additionally, further verification of 2-ME2S and CS compound identity is still required via sulfate synthesis, MS/MS fragmentation, and retention time matching.

## 4. Materials and Methods

### 4.1. Chemicals and Reagents

Ammonium formate (99.995%), diethylamine (DEA), ethyl acetate (EtOAc), methanol (MeOH), and sodium hydroxide (NaOH) of LC-MS grade were purchased from Merck (Kilsyth, Australia). The water used was ultrapure grade (18.2 MΩ·cm) obtained from a ThermoFisher Barnstead Smart2Pure system (ThermoScientific; Langenselbold, Hungary).

### 4.2. Administration Study

This research utilized samples collected from 6 horses (5–14 years; 488 ± 49 kg) from a study administering oral (*n* = 3) and IM (*n* = 3) routes of ALT. The amount of ALT administered was the advised therapeutic dosage on the product labels, which is dependent on the total body weight (BW) of the individual horse. Each oral administration dose was 0.044 mg/kg BW from a 2.2 mg/mL oral solution, whereas each IM administration dose was 0.3 mg/kg BW from a 50 mg/mL injectable solution. This study administered daily oral doses for 14 days and two long-acting IM injections of ALT into the muscle at the base of the neck on day zero and at 7 days. Sample collection was conducted at 8 am daily, inclusive of the two weeks of administration, with an additional one week of sampling conducted after the conclusion of administration, totaling 21 days of sample collection. Two frequent sampling days were also conducted at the first and final administration points for each route where samples were collected every 2 h until the 8-h mark, plus an additional 12-h sample. The individual frequent sample days were day zero and day 14 for oral and day zero and day 7 for IM administration groups. Samples were stored at −20 °C prior to sample extraction. Ethics approval was provided for this study by Charles Sturt University (A19050, Wagga Wagga, NSW, Australia). Further information regarding the administration study conditions can be found in the Supplementary Material (Section S3).

### 4.3. Sample Preparations and Instrumental Analyses 

The untargeted steroid sulfate extraction and analysis was adapted from a method developed and published by Fitzgerald et al. [26,27]. Briefly, urine aliquots (0.6 mL) were diluted with phosphate buffer (0.3 mL, 100 mM, pH 5.4) and centrifuged at 2093 g for 10 min. The supernatant was then combined with an internal standard mix (testosterone sulfate-d_3_, epitestosterone glucuronide-d_4_, pregnenolone-d_4_, trenbolone-d_5_, progesterone-d_9_; 20 µL) in a fresh Eppendorf tube, before being loaded into a solid phase extraction (SPE) weak anion exchange (WAX) cartridge (from Waters, Rydalmere, Australia) conditioned with MeOH (3 mL) and ultrapure water (3 mL). The cartridge was then consecutively washed with NaOH (2 mL, 0.1 M), phosphate buffer (3 mL, 100 mM, pH 5.4), and ultrapure water (3 mL) before steroid sulfates were eluted with a solution of EtOAc: MeOH: DEA (3 mL, 25:25:1% *v*/*v*). The extract was transferred into spin filters (from PhaseSep, Doncaster East, Australia) before being dried under nitrogen at 60 °C and reconstituted with 10% MeOH in ultrapure water (0.25 mL). Extracted samples were stored at 4 °C prior to instrument analysis. 

Samples were analyzed using a Shimadzu LC-40 ultra-high-performance liquid chromatography coupled to a 9030-quadrupole time of flight-mass spectrometer (UHPLC-QTOF-MS). The column was a Shim-pack Velox C-18 (1.8 μm, 2.1 mm × 100 mm, Shimadzu, Kyoto, Japan). Mobile solutions included A: 20 mM ammonium formate in 100% ultrapure water and B: 20 mM ammonium formate in 100% MeOH. This method utilized an elution gradient of 0–0.50 min (10% B), 0.51–20.50 min (10–100% B), 20.51–23.00 min (100% B), and 23.01–27.50 min (10% B). The flow rate was 0.4 mL/min, the injection volume used was 5 µL, and the column temperature was 40 °C. Data-dependent acquisition (DDA) analysis was performed in positive and negative electrospray ionization (ESI) modes. MS conditions included a full scan range of *m*/*z* 100–1000 and MS/MS range of *m*/*z* 50–1000 with an intensity threshold of 500. The collision energy (CE) spread utilized was 60 ± 15 eV, with a 2.50 kV spray voltage, resolution of 30,000 FWHM, and approximately 19 Hz scan speed.

### 4.4. Statistical Multiplatform Workflow

The processing method was adapted from C. Fitzgerald et al. [26,27]. Further information regarding the workflow can be found in the appropriate references, cited above. Sample data was exported from LabSolutions (version 4.50 SP1) in the raw file format (.lcd) and imported to MS-DIAL (version 4.90) where it was processed, aligned, and normalized (LOWESS). This resulted in the generation of a Microsoft Excel (version 16.42) spreadsheet containing the normalized peak area intensities for all present features in a batch. This was manually refined by true MS/MS collection, signal-to-noise ratio (S/N), and minimum presence in batch (denoted by Fill%) thresholds before being imported to R for statistical analysis. Simultaneously, the raw LC files (converted to .mgf file format) were run through two Python scripts, using Jupyter Notebook (version 6.3.0), that extracted the MS/MS peak data from each file and amalgamated all the data into one spreadsheet. This data was then carried into RStudio (RStudio–version 2022.07.1, R–version 4.3.2), and statistical analysis was performed with a custom R script. A steroid sulfate searcher function was also created and utilized with the simple ‘if else’ request from the dplyr package against a prearranged list of 26 potential *m*/*z* of interest. This element of the script resulted in information collection of possible significant steroid sulfate peaks. 

In addition to the above workflow, the results from the steroid sulfate searcher function were analyzed in MetaboAnalyst 6.0 (https://www.metaboanalyst.ca, accessed on 3 September 2024) using the Biomarker Analysis tool to determine key features for differentiation between the oral and IM groups. A random forest classification model was then tested on five selected features. 

## 5. Conclusions

A metabolomic approach to the differentiation of two equine administration routes of the steroidal progestin altrenogest was investigated. Liquid chromatography high-resolution mass spectrometry analysis of equine urine samples found five sulfated compounds with the potential to differentiate between oral and intramuscularly injected altrenogest administration using a random forest classification model. Using estrone sulfate, testosterone sulfate, 2-methoxyestradiol sulfate, pregnenolone sulfate, and cortisol sulfate, the best model results gave an AUC score of 0.965 with a confidence level of 95% (between 0.931 and 0.995). These compound identifications were confirmed with assistance from the Shimadzu Insight Explore Assign feature, as well as MS/MS spectrum and retention time matching with purchased and synthesized reference standards.

## Figures and Tables

**Table 1:** Results obtained from the steroid sulfate searcher function included in the statistical processing R script.

	***m*** **/*z***	**RT (min)**
(1)	349.11209	9.333
(2)	367.15909	10.108
(3)	381.13840	8.462
(4)	395.19061	12.454
(5)	441.15961	4.963

**Table 2:** Putatively identified compounds with chemical formulas and structural information.

	Compound	Formula	Structure
(1)	Estrone Sulfate (E1S)	C_18_H_22_O_5_S	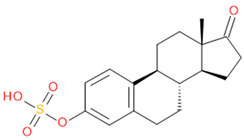
(2)	Testosterone Sulfate (TS)	C_19_H_28_O_5_S	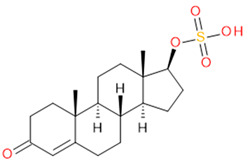
(3)	2-Methoxy-Estradiol Sulfate (2-ME2S)	C_19_H_26_O_6_S	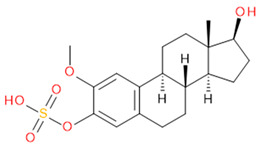
(4)	Pregnenolone Sulfate (PregS)	C_21_H_32_O_5_S	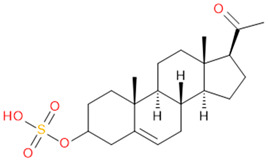
(5)	Cortisol Sulfate (CS)	C_21_H_30_O_8_S	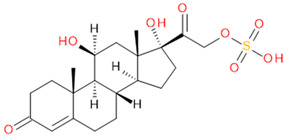

**Table 3:** Univariate receiver operating characteristics (ROC) curve statistical results output from the MetaboAnalyst Biomarker Analysis function. Statistical information (round down to three significant figures) is listed as AUC (area under the curve) scores, *p*-values (also titled *t*-test results in MetaboAnalyst Report), Log_2_FC (log fold change), and cluster identifier numbers denoting which features are clustering together/considered to behave in a similar manner in the dataset.

	AUC	*p*-Value	Log_2_FC	Clusters
2-ME2S	0.840	1.70 × 10^−17^	1.15	1
TS	0.657	2.82 × 10^−5^	−0.936	4
E1S	0.620	9.62 × 10^−5^	−3.35	3
CS	0.550	5.05 × 10^−1^	0.109	2
PregS	0.538	7.95 × 10^−2^	−3.05	4

**Table 4:** Confusion matrix results from the multivariate ROC curve. The dataset consisted of 183 samples, with 93 IM administration samples (positive outcome) and 90 oral administration samples (negative outcome).

		Predicted	
		Positive (IM)	Negative (Oral)
**Actual**	**Positive (IM)**	83	10
	**Negative (Oral)**	15	75

## Data Availability

The raw data supporting the conclusions of this article will be made available by the authors upon request.

## Supplementary Materials

The following supporting information can be downloaded at: https://www.mdpi.com/article/10.3390/molecules29214988/s1, Table S1. HRMS (-ESI) details for obtained synthesized reference compounds; Table S2. Sampling regimen for the oral administration of altrenogest; Table S3. Sampling regimen for the intramuscular administration of altrenogest; Figure S1. Classical ROC curve and box plot for 2-methoxy-estradiol sulfate (2-ME2S); Figure S2. Classical ROC curve and box plot for testosterone sulfate (TS); Figure S3. Classical ROC curve and box plot for estrone sulfate (E1S); Figure S4. Classical ROC curve and box plot for cortisol sulfate (CS); Figure S5. Classical ROC curve and box plot for pregnenolone sulfate (PregS); Figure S6. Multivariate AUROC using a random forest algorithm model; Figure S7. Model results of best two features; Figure S8. Model results of best three features; Figure S9. Model results of best four features; Figure S10. Model results of all five features; Figure S11. Predictive accuracy results for all combinations of features tested by the model. References [26,27] are cited in the Supplementary Materials.
