# Supplementary material for: Administration Route Differentiation of Altrenogest via the Metabolomic LC-HRMS Analysis of Equine Urine
Source: Molecules. 2024 Oct 22;29(21):4988. doi: 10.3390/molecules29214988 (PMC11547534; doi:10.3390/molecules29214988)
Supplement: Supplementary file 1 [file molecules-29-04988-s001.zip › Supplementary Materials_accepted.pdf]

Supplementary Materials

Section 1. Compound Identification

Table S1 provides the found *m/z*, retention time (RT) and some characteristic MS/MS fragmentation observed from synthesised estrone sulfate, testosterone sulfate, and pregnenolone sulfate, provided by Christopher C.J Fitzgerald. These compounds were analysed neat (without urine matrix) to provide MS/MS, a small amount of RT shift can, therefore, be expected. Detailed methodology of the synthesis of these compounds and similar compound types has been previously published and can be obtained from the referenced articles' supplementary material [1,2].

Table S1. HRMS (-ESI) details for obtained synthesized reference compounds.

	Reference Compound	Structure	Found *m/z* and RT (min)	(-)ESI MS/MS (*m/z*)
(1)	Estrone Sulfate<br>(E1S)<br>$C_{18}H_{22}O_5S$	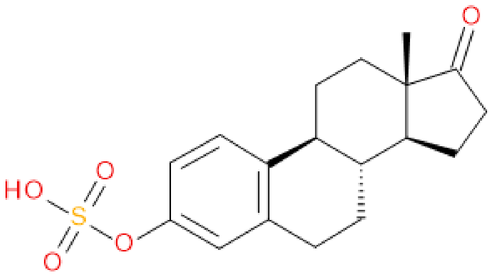	m/z 349.1106, 9.203 min	81.21106<br>145.06341<br>183.73195<br>269.15411<br>349.11068
(2)	Testosterone Sulfate<br>(TS)<br>$C_{19}H_{28}O_5S$	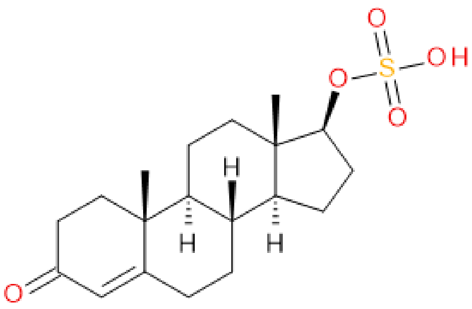	m/z 367.1576, 10.090 min	79.95511<br>96.95901<br>177.02073<br>273.87398<br>352.13628<br>367.15753
(4)	Pregnenolone Sulfate<br>(PregS)<br>$C_{21}H_{32}O_5S$	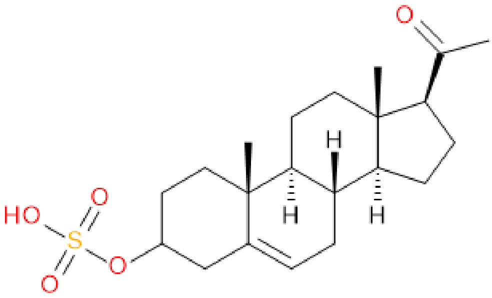	m/z 395.1888, 12.454 min	80.41009<br>96.95900<br>271.53139<br>290.36310<br>395.18897

Section 2. MetaboAnalyst Parameters

Classical ROC curve analysis of individual features with AUC scores and 95% confidence level in parentheses on plot. Boxplots to the right indicate the abundance spread of each individual feature in the oral (0) and IM (1) groups, respectively.

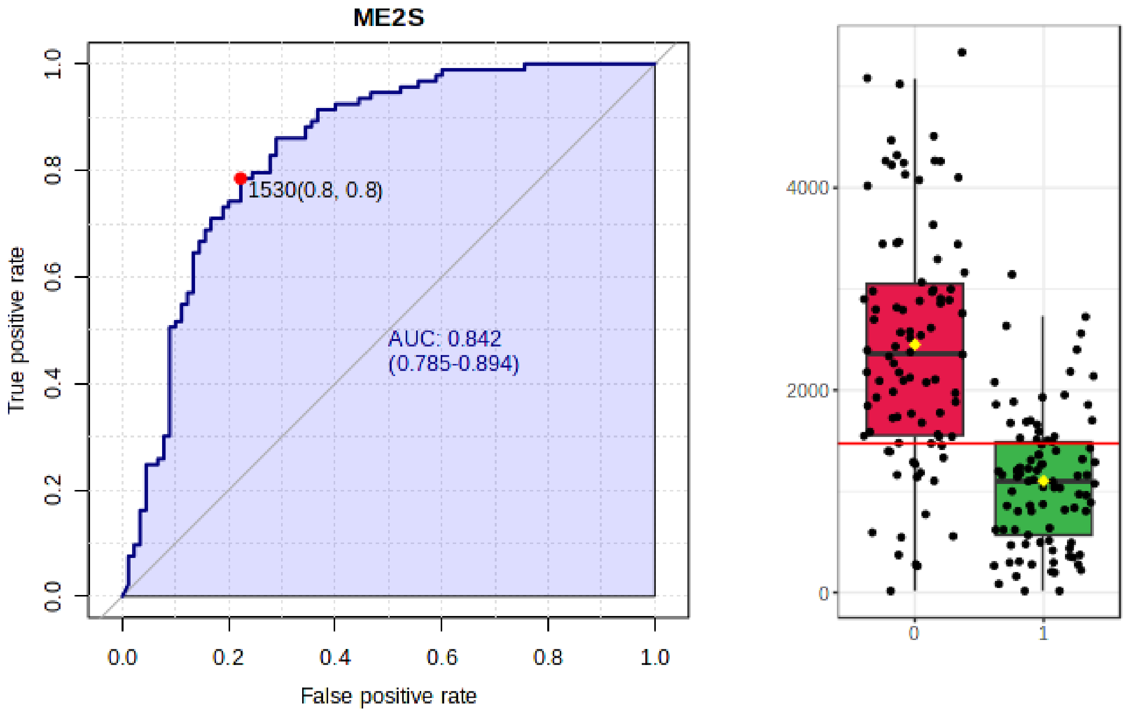

Figure S1. Classical ROC curve and box plot for 2-methoxy-estradiol sulfate (2-ME2S).

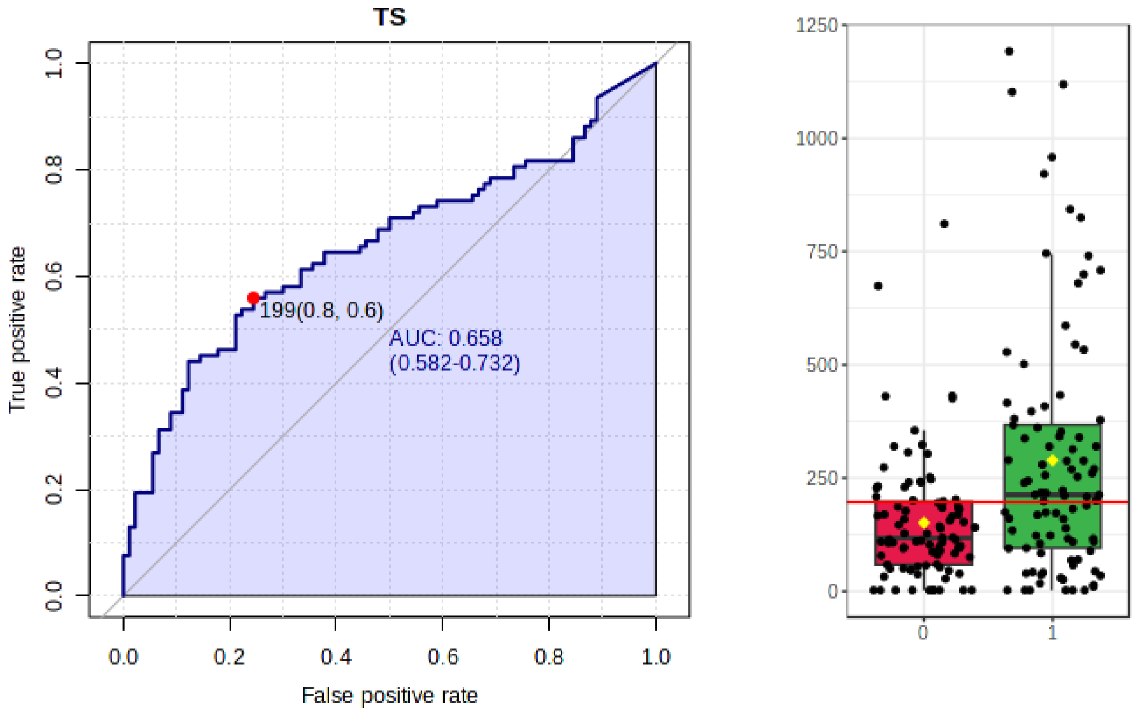

Figure S2. Classical ROC curve and box plot for testosterone sulfate (TS).

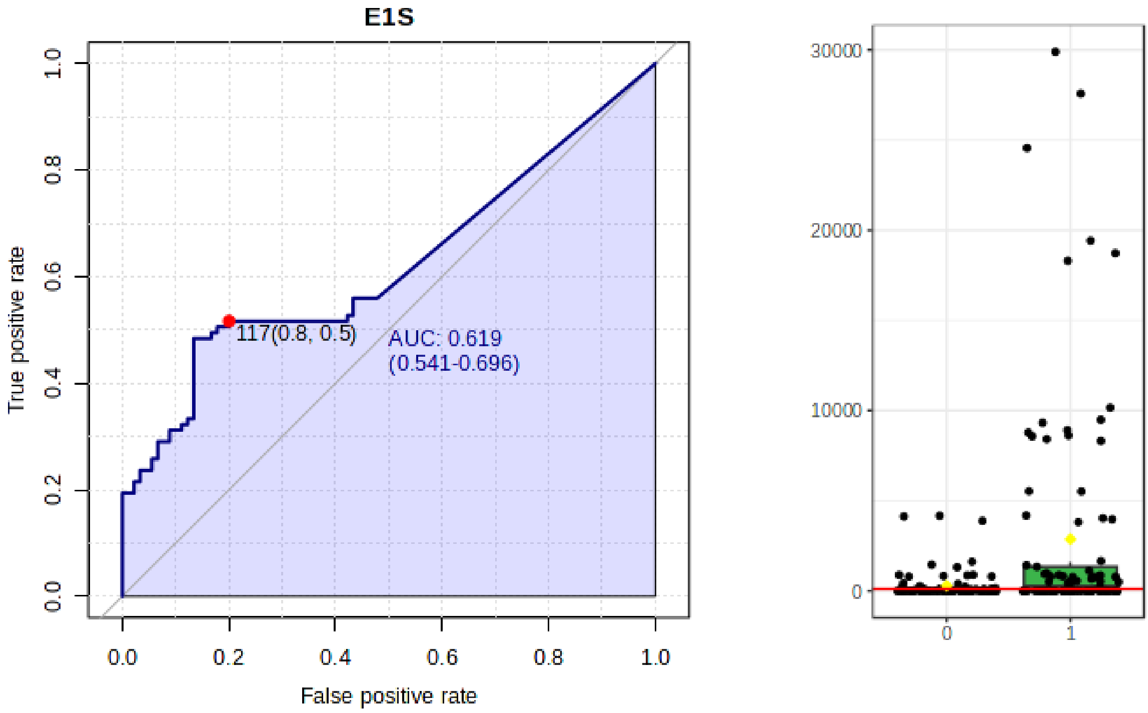

Figure S3. Classical ROC curve and box plot for estrone sulfate (E1S).

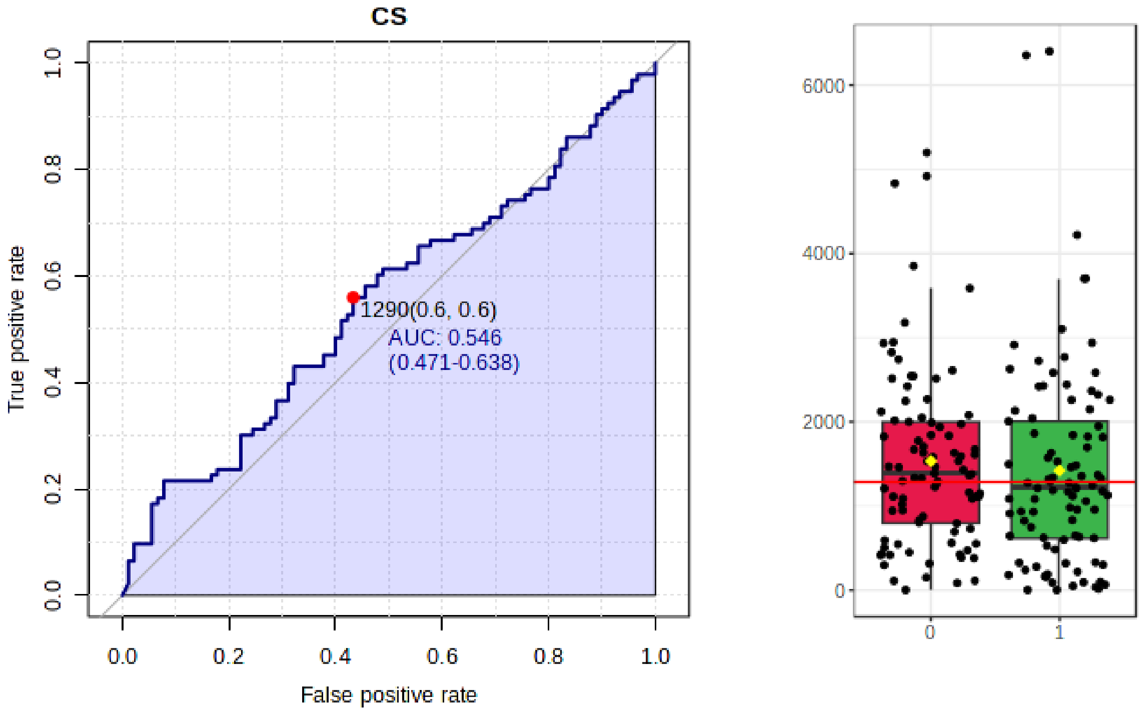

Figure S4. Classical ROC curve and box plot for cortisol sulfate (CS).

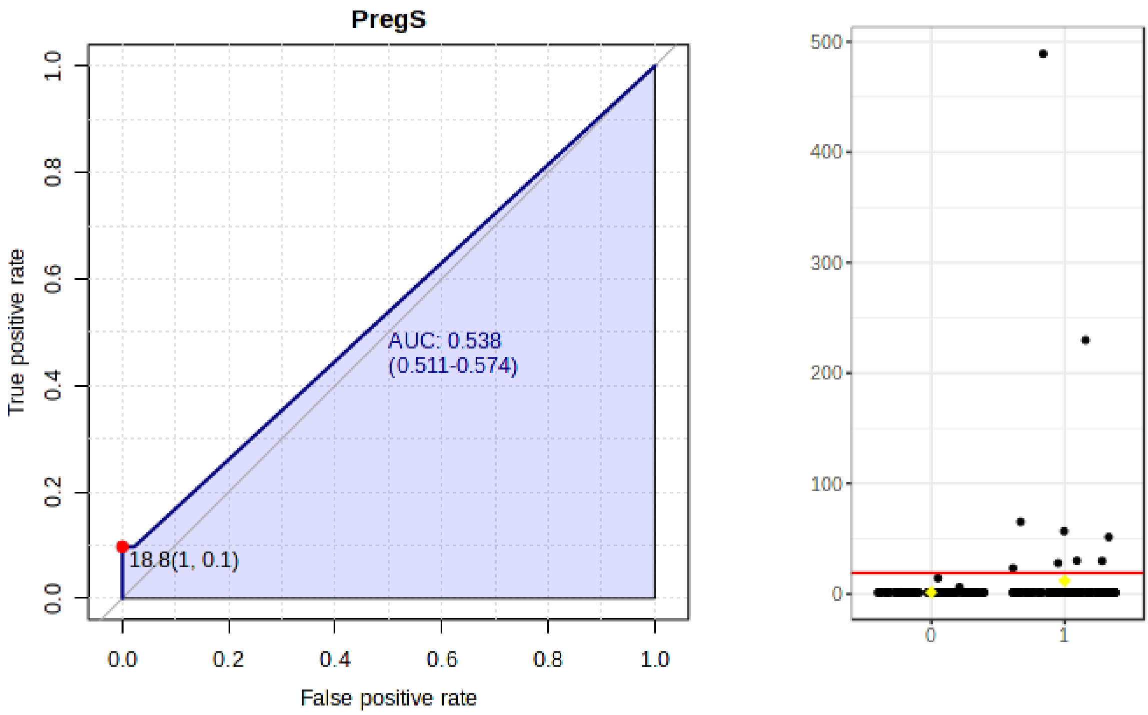

Figure S5. Classical ROC curve and box plot for pregnenolone sulfate (PregS).

Figure S6 shows a plot of ROC curves for all biomarker models tested (Var. indicates how many features included in model test) based on its average performance across all MCCV runs using the random Forests algorithm provided in MetaboAnalyst 6.0. Figures S7-10 present the same ROC curve plots individually.

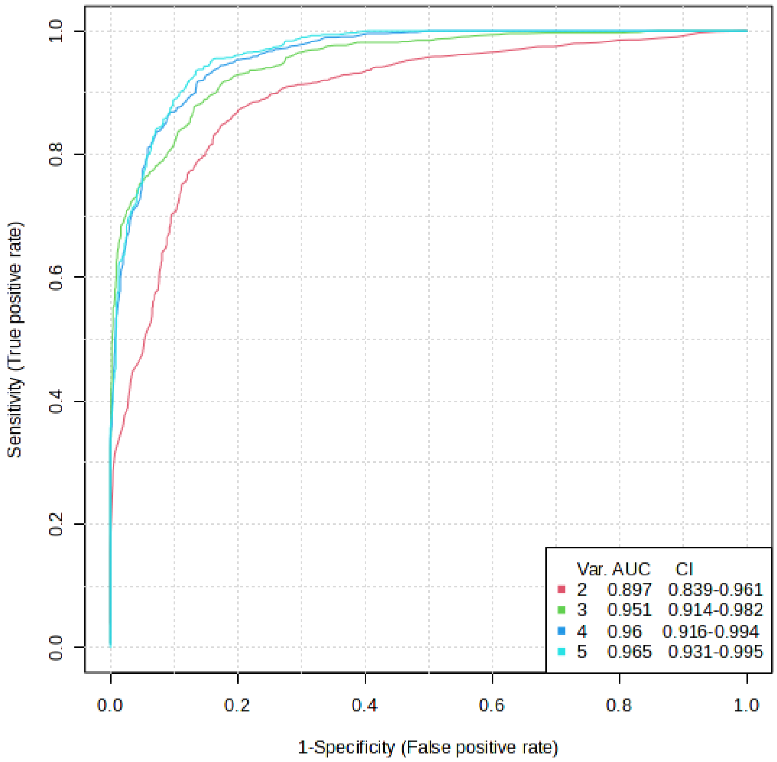

Figure S6. Multivariate AUROC using a random forest algorithm model.

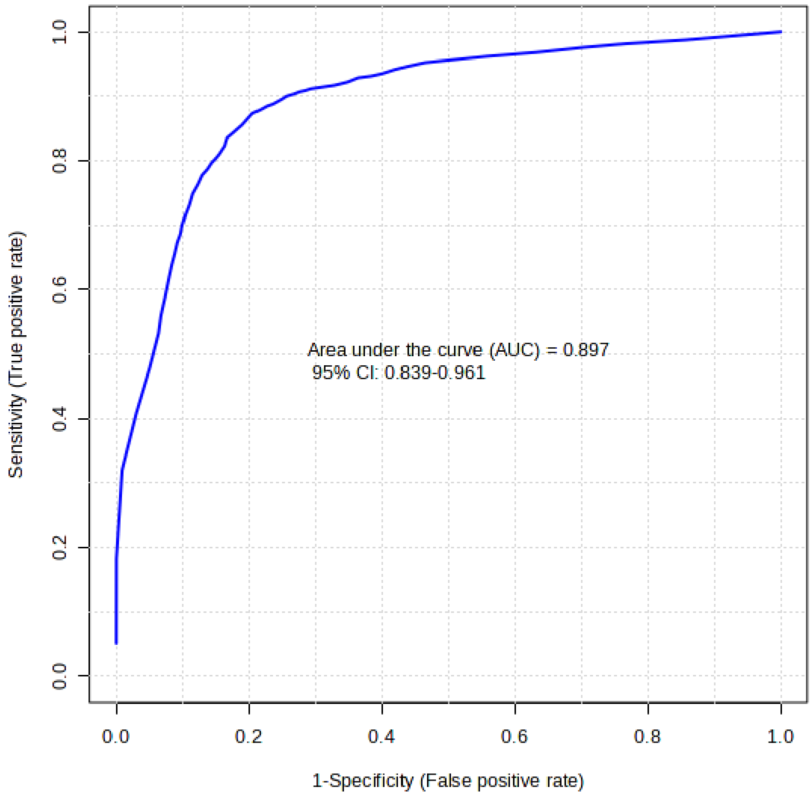

Figure S7. Model results of best two features.

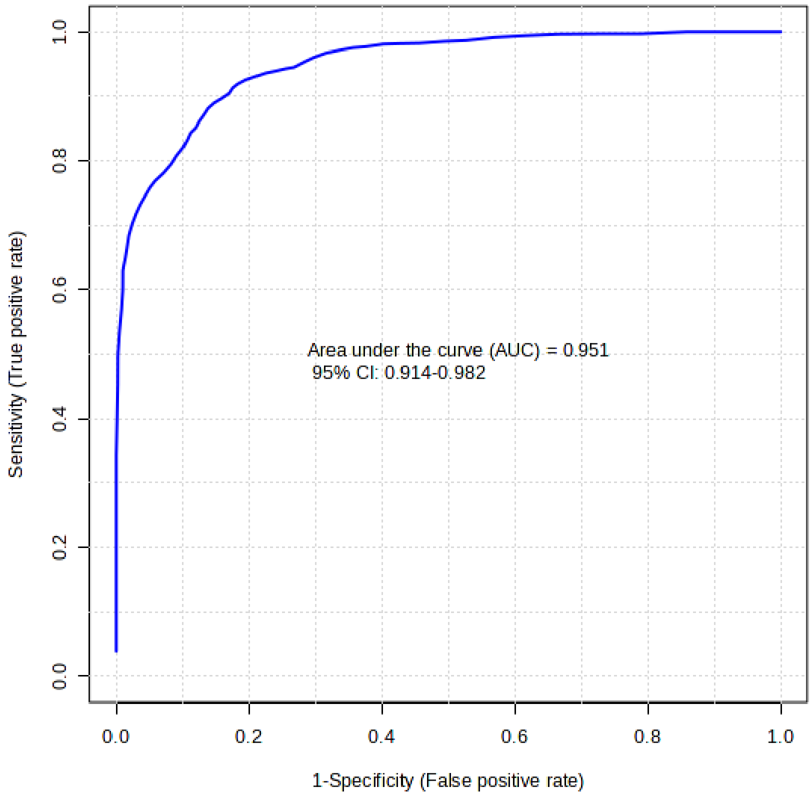

Figure S8. Model results of best three features.

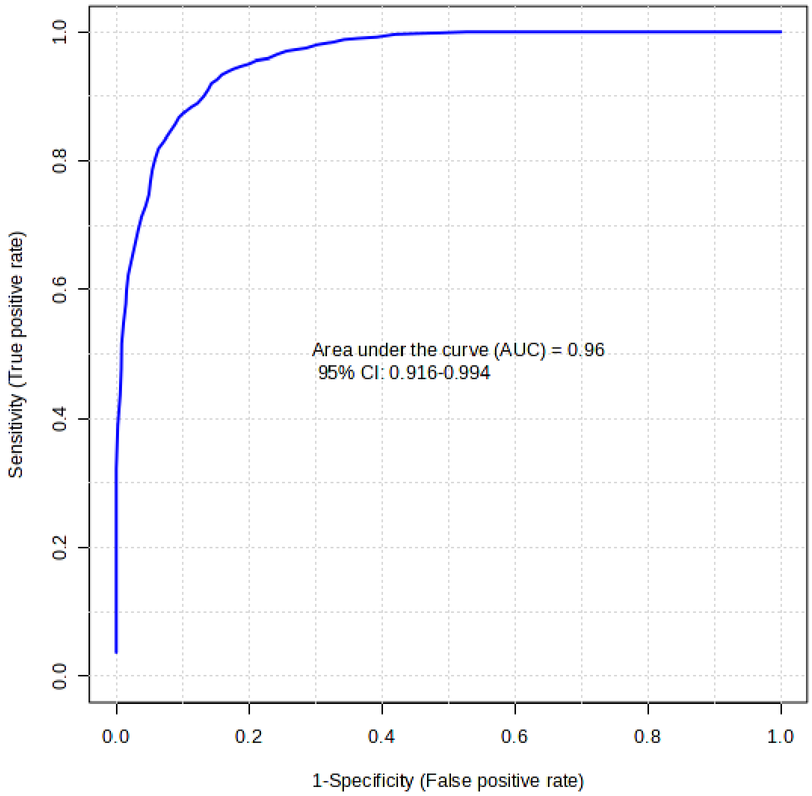

Figure S9. Model results of best four features.

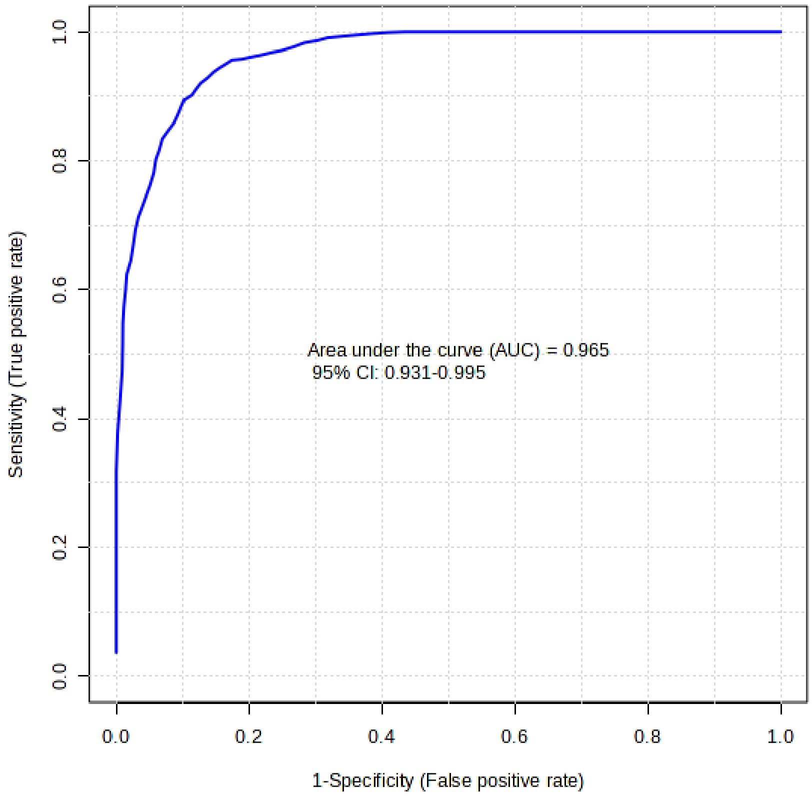

Figure S10. Model results of all five features.

Figure S11 shows the predictive accuracy of each biomarker model with an increasing number of features. The biomarker model with all five features included (highlighted by a red dot) gave the highest predictive accuracy at 86.7%.

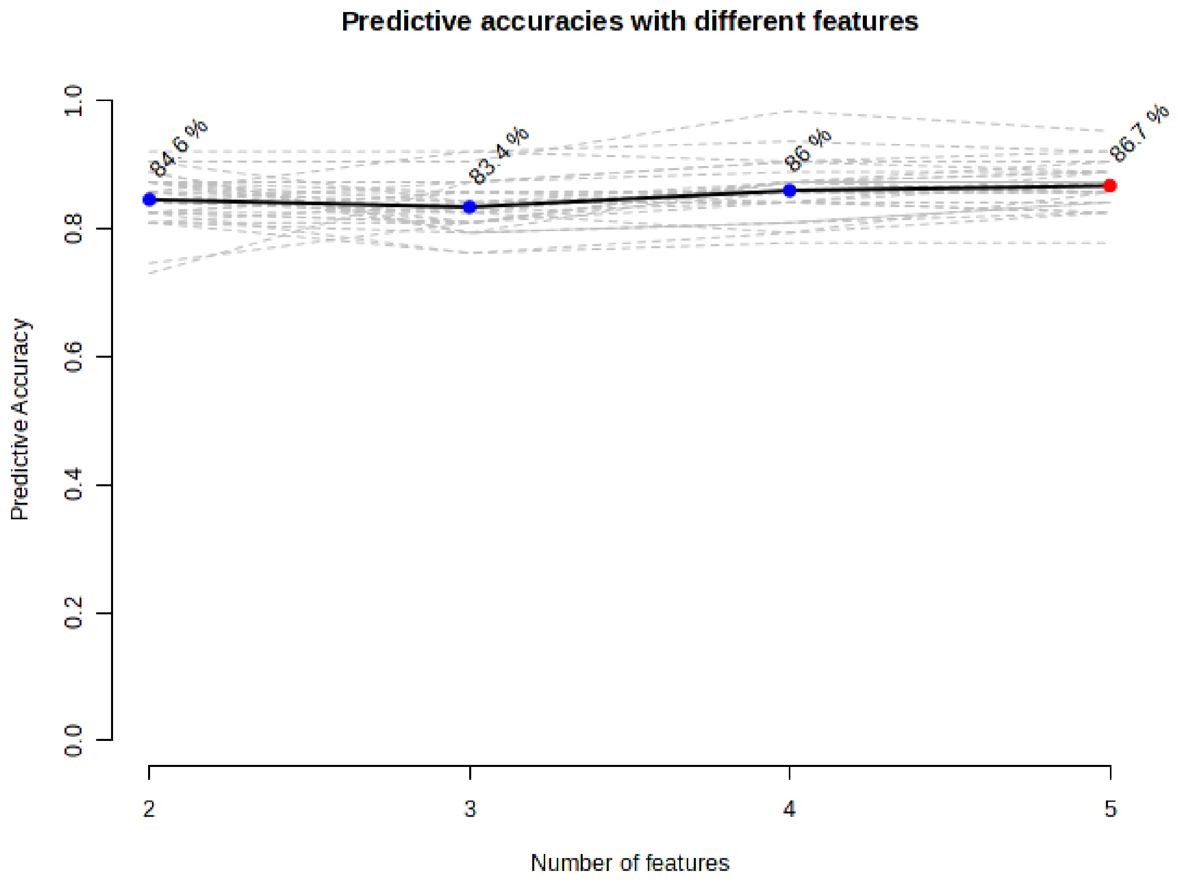

Figure S11. Predictive accuracy results for all combinations of features tested by the model.

Section 3. Additional Administration Study Information

Each administration horse had their heart rate, respiratory rate, rectal temperature and bodyweight (BW) manually measured prior to the study commencing to check normal health. Each horse was assigned a stable of yard for the duration of the sampling and fed meals of their regular (unchanged) hay ration of approximately 2 % BW dry matter, and access to clean drinking water as all times. All horses were allowed free exercise in an adjacent paddock to their assigned stable/yard for a minimum of 1 hour each day.

Urine sampling commenced at 7 am on day zero (pre-administration, time zero), and dosing of oral and IM ALT occurred at 8 am. Oral dosing occurred each day at 8 am after first administration, with each 24-hour urine sample being collected at 8 am (Table S2 and Table S3)

Table S2. Sampling regimen for the oral administration of altrenogest.

**Day**	**Time (approx.)**	**Oral Admin**<br>**Urine collection time points**
**Day 0**	**from 7am**	**Pre Admin (T0)**
Dose horses with ALT orally	8 am	-
	10 am	2 h
	12 pm	4 h
	2 pm	6 h
	4 pm	8 h
	8 pm	12 h
**1**	8 am	24 h
**2**	8 am	48 h
**3**	8 am	72 h
**4**	8 am	96 h
**5**	8 am	120 h
**6**	8 am	144 h
**7**	8 am	168 h
**8**	8 am	192 h
**9**	8 am	216 h
**10**	8 am	240 h
**11**	8 am	288 h
**12**	8 am	312 h

**Day 13 End of dosing elimination profile**	**from 7am**	**336 h**
Final oral ALT dose	8 am	-
	10 am	338 h
	12 pm	340 h
	2 pm	342 h
	4 pm	344 h
	8 pm	348 h
**14**	8 am	360 h
**15**	8 am	384 h
**16**	8 am	408 h
**17**	8 am	432 h
**18**	8 am	456 h
**19**	8 am	480 h
**20**	8 am	504 h

Table S3. Sampling regimen for the intramuscular administration of altrenogest.

**Day**	**Time (approx.)**	**IM Admin**<br>**Urine collection time points**
**Day 0**	**from 7am**	**Pre Admin (T0)**
Dose horses with ALT IM	8 am	-
	10 am	2 h
	12 pm	4 h
	2 pm	6 h
	4 pm	8 h
	8 pm	12 h
**1**	8 am	24 h
**2**	8 am	48 h
**3**	8 am	72 h
**4**	8 am	96 h
**5**	8 am	120 h

**6**	8 am	144 h
**Day 7**<br>**Second dosing elimination profile**	**from 7am**	**168 h**
Second (final) ALT IM dose	8 am	-
	10 am	170 h
	12 pm	172 h
	2 pm	174 h
	4 pm	176 h
	8 pm	180 h
**8**	8 am	192 h
**9**	8 am	216 h
**10**	8 am	240 h
**11**	8 am	288 h
**12**	8 am	312 h
**13**	8 am	336 h
**14**	8 am	360 h
**15**	8 am	384 h
**16**	8 am	408 h
**17**	8 am	432 h
**18**	8 am	456 h
**19**	8 am	480 h
**20**	8 am	504 h

References:

1. Fitzgerald, C.C.J.; Hedman, R.; Uduwela, D.R.; Paszerbovics, B.; Carroll, A.J.; Neeman, T.; Cawley, A.; Brooker, L.; McLeod, M.D. Profiling Urinary Sulfate Metabolites With Mass Spectrometry. *Front Mol Biosci* **2022**, *9*, 829511, doi:https://doi.org/10.3389/fmolb.2022.829511.
2. Fitzgerald, C.C.J.; Bowen, C.; Elbourne, M.; Cawley, A.; McLeod, M.D. Energy-Resolved Fragmentation Aiding the Structure Elucidation of Steroid Biomarkers. *Journal of the American Society for Mass Spectrometry* **2022**, *33*, 1276-1281, doi:https://doi.org/10.1021/jasms.2c00092.
