# Supplementary figures and images for: Administration Route Differentiation of Altrenogest via the Metabolomic LC-HRMS Analysis of Equine Urine
Source: Molecules. 2024 Oct 22;29(21):4988. doi: 10.3390/molecules29214988 (PMC11547534; doi:10.3390/molecules29214988)

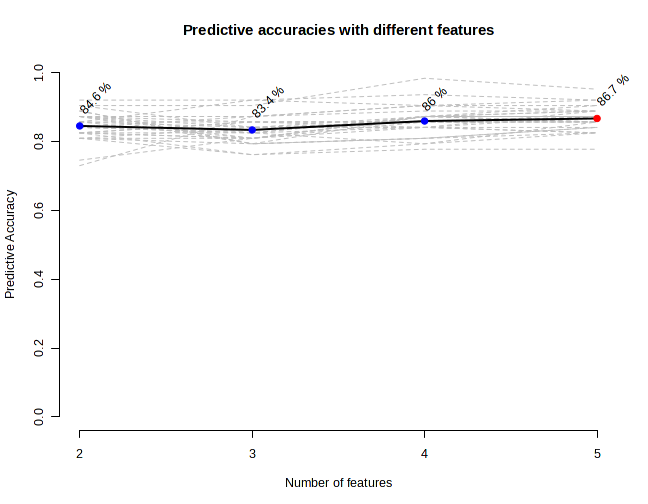

Supplement: Supplementary file 1 [file molecules-29-04988-s001.zip › cls_accu_1_dpi72.png]

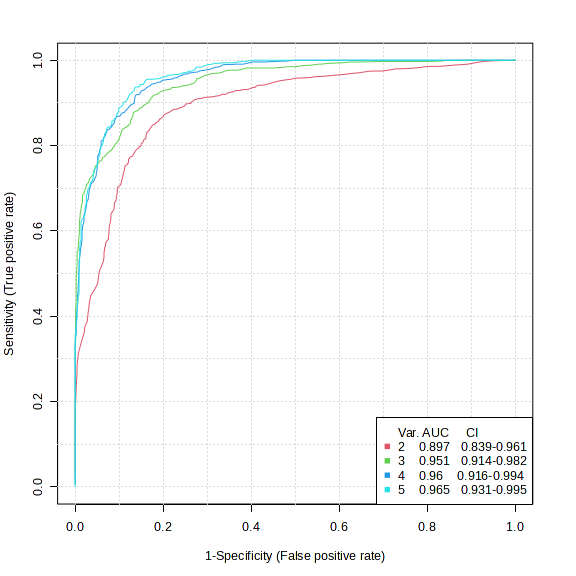

Supplement: Supplementary file 1 [file molecules-29-04988-s001.zip › cls_roc_0_dpi72.png]

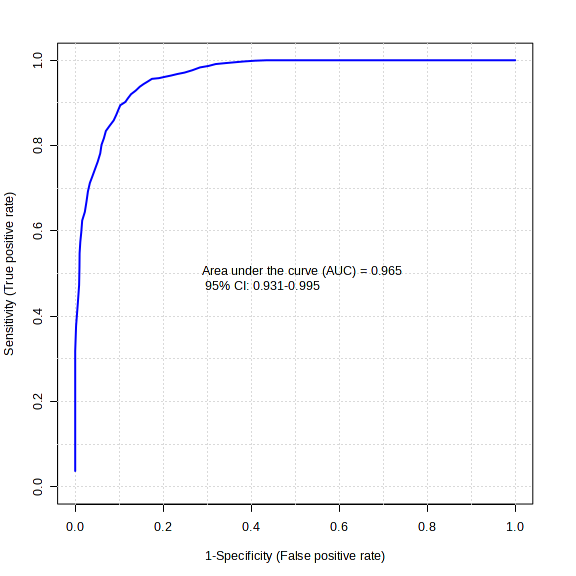

Supplement: Supplementary file 1 [file molecules-29-04988-s001.zip › cls_roc_1_dpi72.png]

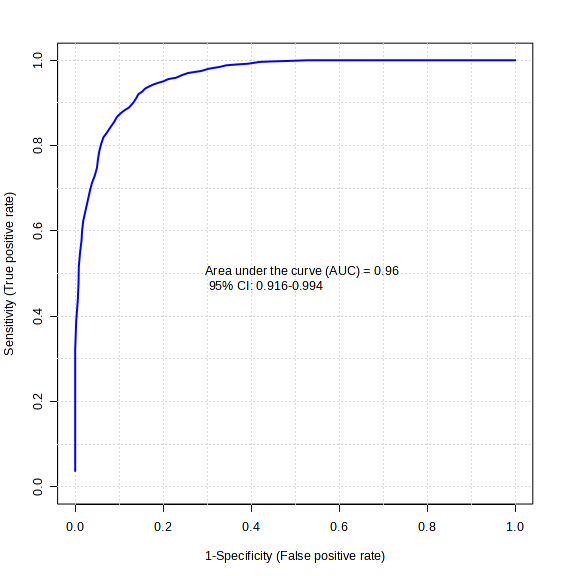

Supplement: Supplementary file 1 [file molecules-29-04988-s001.zip › cls_roc_2_dpi72.png]

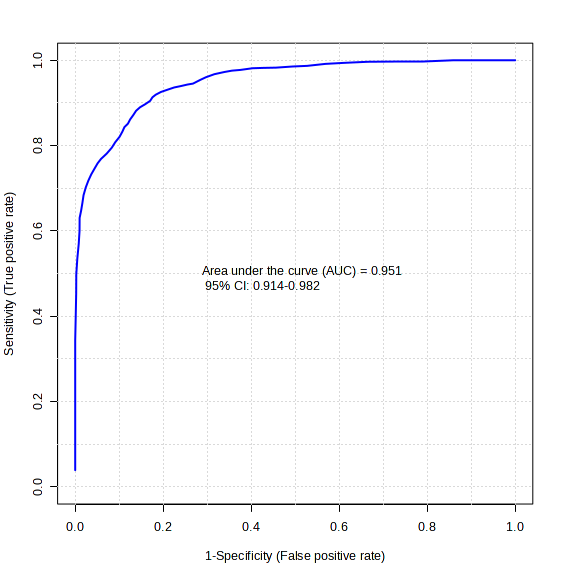

Supplement: Supplementary file 1 [file molecules-29-04988-s001.zip › cls_roc_3_dpi72.png]

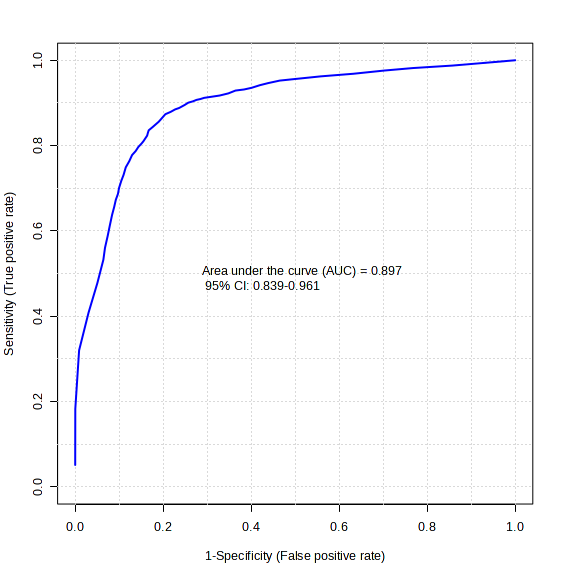

Supplement: Supplementary file 1 [file molecules-29-04988-s001.zip › cls_roc_4_dpi72.png]

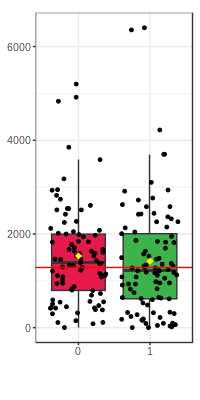

Supplement: Supplementary file 1 [file molecules-29-04988-s001.zip › roc_boxplot_CS_5_dpi72.png]

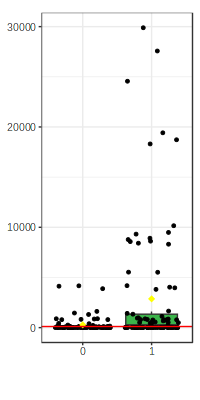

Supplement: Supplementary file 1 [file molecules-29-04988-s001.zip › roc_boxplot_E1S_4_dpi72.png]

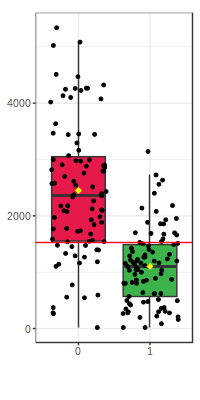

Supplement: Supplementary file 1 [file molecules-29-04988-s001.zip › roc_boxplot_ME2S_2_dpi72.png]

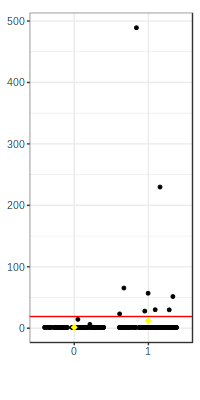

Supplement: Supplementary file 1 [file molecules-29-04988-s001.zip › roc_boxplot_PregS_6_dpi72.png]

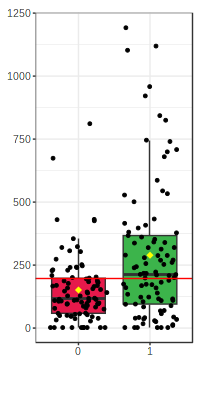

Supplement: Supplementary file 1 [file molecules-29-04988-s001.zip › roc_boxplot_TS_3_dpi72.png]

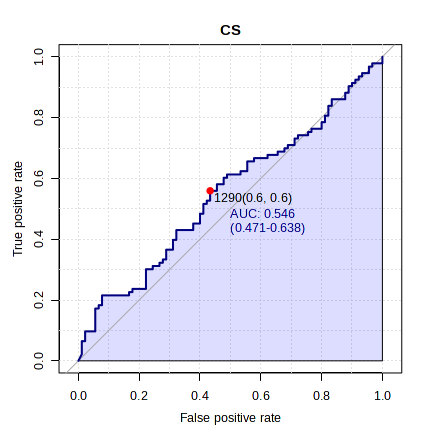

Supplement: Supplementary file 1 [file molecules-29-04988-s001.zip › roc_univ_CS_5_dpi72.png]

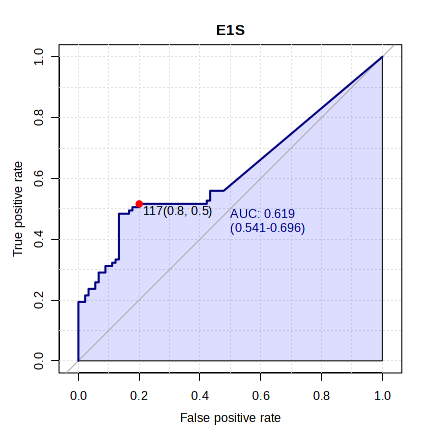

Supplement: Supplementary file 1 [file molecules-29-04988-s001.zip › roc_univ_E1S_4_dpi72.png]

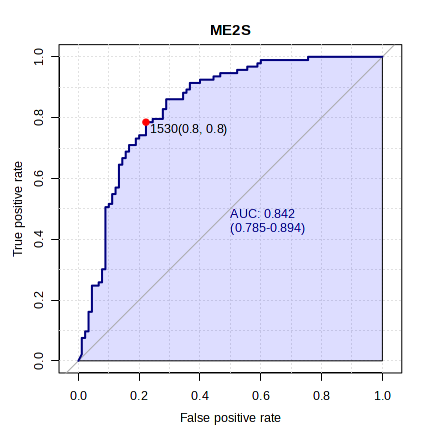

Supplement: Supplementary file 1 [file molecules-29-04988-s001.zip › roc_univ_ME2S_2_dpi72.png]

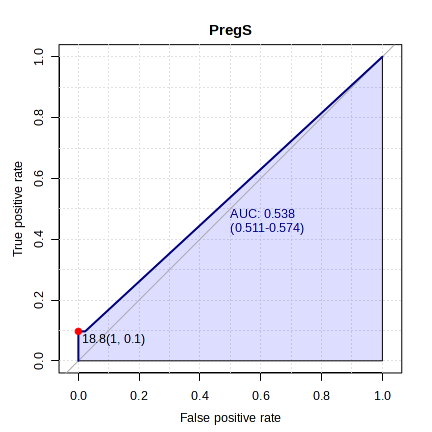

Supplement: Supplementary file 1 [file molecules-29-04988-s001.zip › roc_univ_PregS_6_dpi72.png]

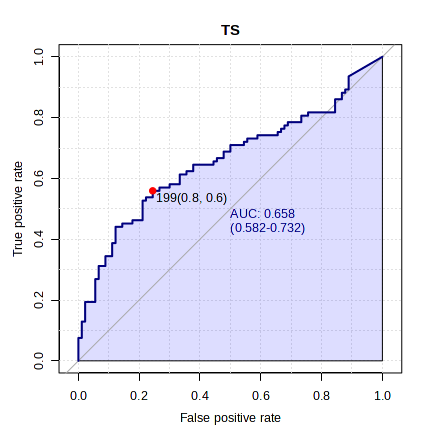

Supplement: Supplementary file 1 [file molecules-29-04988-s001.zip › roc_univ_TS_3_dpi72.png]
